# Action Contribution to Competence Judgments: The Use of the Journey Schema

**DOI:** 10.3389/fpsyg.2016.00448

**Published:** 2016-03-30

**Authors:** Oleksandr V. Horchak, Jean-Christophe Giger, Margarida V. Garrido

**Affiliations:** ^1^CIS-IUL, Instituto Universitário de Lisboa (ISCTE-IUL)Lisboa, Portugal; ^2^Department of Psychology and Educational Sciences, University of AlgarveFaro, Portugal; ^3^Research Centre for Spatial and Organizational Dynamics — CIEO, University of AlgarveFaro, Portugal

**Keywords:** embodied cognition, metaphor, competence, warmth, politician perception

## Abstract

The current research considered the question of how performing an action, or merely preparing the body for action, can have an impact on social judgments related to person perception. Participants were asked to ascribe competence and warmth characteristics to a target person by reading a metaphoric text while their body was manipulated to be prepared for the processing of action-congruent information. In Experiment 1, participants whose forward body action matched the metaphoric action described in the text ascribed more competence characteristics to a politician than did control participants. In Experiment 2, participants whose body was merely prepared for forward movement also ascribed more competence characteristics to a politician than did control participants. In addition, the data from Experiment 2 ruled out an alternative non-embodied explanation (i.e., that effect is due to basic associative processes) grounded in the existing literatures on attitudes by demonstrating that body manipulation had no effect on competence when a non-metaphoric text was used. Finally, no evidence was found that body manipulation affects warmth judgments. These studies converge in demonstrating that forward body movements enhance the favorability of competence judgments when these match the metaphoric forward movements described by text.

## Introduction

Consider the following two extracts from political speeches in which a politician outlines before the audience the ways of building a better society.

### Extract A

By protecting the life of our nation and reserving the liberties of our citizens we pursue our own happiness. Our success in that pursuit is the test of our success as a nation. If we have a false sense of independence, in the journey to the better tomorrow our ships can collide and crash. But if we find commitment to new priorities, to new strategies, and new ways of thinking that ensure that hope will be kept alive, we will break the wall of hesitation and safely navigate our vessel to the better future.

### Extract B

By protecting the life of our nation and reserving the liberties of our citizens we ensure our own happiness. Our success in that task is the test of our success as a nation. If we have a false sense of independence, in the desire to change the future we may get disappointed. But if we find commitment to new priorities, to new strategies, and new ways of thinking that ensure that hope will be kept alive, we will no longer hesitate and achieve great success in our objectives.

Extract A and Extract B differ in that A describes the process of achieving a purpose in metaphorical terms as “purpose is a destination.” Why is it that people often talk about such abstract concepts as “purposes” or “progress” in terms of bodily actions like “move forward,” “move ahead,” or “forge ahead?” Conceptual Metaphor Theory (CMT) of Lakoff and Johnson (1980) suggests that this happens because mental representations of abstract concepts are grounded in sensory-motor (embodied) experiences. More precisely, this theory posits that we represent abstract concepts in terms of concrete concepts by metaphorical mapping, when the terms of the source domain (e.g., destination or journey) are translated into the corresponding terms of the target domain (e.g., purpose or progress).

According to Landau and Keefer (2014), such metaphors of communication when an abstract concept is compared to an unrelated concrete concept (i.e., conceptual metaphors) pervade public political discourse and help politicians to convince the audience of their political claims. This view is further supported by a meta-analysis study of Sopory and Dillard (2002) which concludes that the inclusion of metaphor in political discourse is likely to increase persuasion, especially if the metaphor semantically matches other metaphorical phrases contained in the communication. Nonetheless, while all researchers agree that conceptual metaphorical representations facilitate social cognition, there has been a considerable debate about the nature of such metaphorical representations.

## Embodied theory and research

For years, traditional amodal theories assumed that social information processing is based on schematic and disembodied categories. According to this view, the interpretation of metaphorical information is based on amodal symbols that redescribe sensorimotor and affective experiences. This view derived from a number of influential studies (e.g., Fodor, 1975; Pylyshyn, 1984; Landauer and Dumais, 1997) that compared human cognition to computerized processing of information. These studies argued that sensory and motor systems play peripheral roles in information processing, given that their only function boils down to delivering information to a central processing unit (i.e., brain) via sensory systems and executing commands of this unit via action systems.

However, discoveries in cognitive psychology regarding abstract concepts have cast doubt on assumptions of amodal theories. Three main types of evidence supporting an alternative embodied view of cognition exist in the literature. The first and most well-established type of evidence is based on conceptual metaphor theory (Lakoff and Johnson, 1980; Lakoff, 1987) which holds that concepts are understood metaphorically through reference to a more concrete embodied experience. For instance, research in the area of language comprehension has shown that time can be understood in terms of space (e.g., Santiago et al., 2007; Lakens et al., 2011; Sell and Kaschak, 2011), distance in terms of similarity (Boot and Pecher, 2010), number processing in terms of body movements (e.g., Anelli et al., 2014) and categories in terms of containers (Boot and Pecher, 2011). The second type of evidence focuses on the importance of motor system in comprehension. The primary evidence for this approach is the action-sentence compatibility (ACE) effect first reported by Glenberg and Kaschak (2002) who found that reaction times were faster when response direction and the implied direction of concrete or abstract sentences matched, thus suggesting a linkage between motor and linguistic systems. Finally, the third type of evidence demonstrates the involvement of sensory modalities in comprehension of abstract information. For example, Barsalou and Wiemer-Hastings (2005) found that participants tended to associate abstract concepts with social aspects of experience, including simulation and situated action, and concrete aspects with physical entities. These results are exactly what one would expect if participants' comprehension of abstract concepts relied on situated simulations.

The idea of embodied cognition was recognized and examined by many researchers in social cognition, who investigated how metaphors affect attention, memory, attitudes, and perceived social environment. In the domain of attention, Moeller et al. (2008) showed that when individuals were presented with a metaphor “good is up/bad is down,” individual differences in perceived social power had an impact on spatial attention in a metaphor-consistent manner. In the domain of memory, Crawford et al. (2006) showed that individuals remember the metaphor “good is up” better when it is presented in a higher position (see also Palma et al., 2011). In the domain of attitudes, researchers showed that political attitudes can be understood in terms of a horizontal spatial axis (Oppenheimer and Trail, 2010; Farias et al., 2013), morality in terms of cleanliness (Schnall et al., 2008), good and moral in terms of bright color, and bad and immoral in terms of black color (Meier et al., 2007; Sherman and Clore, 2009). Finally, in the domain of perceived social environment, studies revealed that importance can be understood in terms of weight (Jostmann et al., 2009), social power in terms of perceptions of vertical positions (Schubert, 2005), and social exclusion in terms of physical coldness (Zhong and Leonardelli, 2008). For reviews and discussion of many other similar findings that show how various abstract concepts can be understood metaphorically through reference to a more concrete embodied experience, see Borghi and Pecher (2011), Horchak et al. (2014b), Lynott et al. (2013), Semin and Garrido (2015), Semin et al. (2014), and Semin et al. (2012, 2013).

Although, the role of metaphor and embodiment in shaping language processing, attention, memory, attitudes, and perceived social environment is well-documented, much less is known how metaphors influence such an important social psychological phenomenon as personality perception, namely that of a politician. The current study therefore investigates the link between metaphor and embodiment by examining how people's embodied interpretation of language resulting from compatibility between real and metaphoric action influences competence judgments.

## Stereotype content theory and research

Research suggests that when it comes to judging others, people seem to organize their trait knowledge into two dimensions. Some scholars referred to these dimensions as social desirability, or characteristics associated with warmth and trustworthiness, and intellectual desirability, or characteristics associated with competence and efficiency (Rosenberg et al., 1968). Other researchers referred to these dimensions as agency, or characteristics associated with goal achievement and task functioning, and communion, or characteristics associated with maintenance of relationships and social functioning (Abele and Wojciszke, 2007). Still others referred to these dimensions as competence, or characteristics associated with intelligence, skill, creativity, and efficacy, and warmth, or characteristics associated with friendliness, helpfulness, sincerity, trustworthiness, and morality (e.g., Chemers, 2001; Judd et al., 2005; Fiske et al., 2007; Imhoff et al., 2013). In sum, there are various labels for these two fundamental dimensions in the literature. For the sake of clarity, and in order to avoid confusion, from now on we will henceforth use the terms competence to refer to intellectual desirability and agency and warmth to refer to social desirability and communion. These terms were suggested in an influential Stereotype Content Model (SCM) theory by Fiske et al. (2002).

On a general level, SCM holds that competence and warmth govern social judgments across cultures, stimuli, and perceivers. As one example to support their argument, Fiske et al. (2002) refer to the studies from Wojciszke's laboratory which show that competence and warmth account for 82% of the variance in perceptions of social behaviors (e.g., Wojciszke et al., 1998; Wojciszke, 2005). According to Cuddy et al. (2009), throughout history, these two core dimensions emerged as a response to a social need to be able to assess if out-group members intend (i.e., if they are friendly, good-natured, sincere, or warm) or are capable (i.e., if they are confident, skillful, or competent) of harming in-group members. What is all the more noteworthy is that SCM research indicates that most (not all) groups receive ambivalent stereotypes of warmth and competence, as evidenced by high ratings on one dimension and low on the other. In other words, SCM argues that groups are usually stereotyped as competent but cold or as warm but incompetent, therefore suggesting a negative relationship between warmth and competence (Cuddy et al., 2011).

Many of these-just mentioned studies supporting SCM theory further develop the point about the prevalence of two-fold conceptualizations by turning to the effects of non-verbal behaviors on competence and warmth judgments. For example, Cuddy et al. (2011) argue that people exert some control over the impressions they make along the two core dimensions (i.e., competence and warmth) not only through overt behaviors such as, for example, winning a game or getting the best grade on a test, but also through their body language. Specifically, they propose that warmth is conveyed through body movements that indicate positive interest or engagements, such as nodding, leaning forward, or orienting the body toward another person, and competence is conveyed through body movements that indicate dominance and power, such as having an expansive posture (e.g., feet shoulder-width apart). This reasoning was borne out by the data from various experimental studies which revealed that bodily responses such as head movements (e.g., Wells and Petty, 1980), arm flexion vs. arm extension (e.g., Neumann and Strack, 2000), dominant vs. non-dominant hand (Briñol and Petty, 2003), and standing vs. recumbent body posture (e.g., Petty et al., 1983) can affect social judgments, thus suggesting that executing certain body movements can activate concepts implied by those movements.

However, do associations between actions and social judgments imply that warmth and competence can be at least partially embodied? Several lines of research addressed this question. One line of research has suggested that warmth-related abstract concepts such as social distance (avoidance behavior) and social proximity (approach behavior) are tied to concrete bodily experiences. For instance, IJzerman and Semin (2009) showed that perceived social proximity is directly connected to the ambient temperature. More specifically, researchers found that when participants were placed in warm ambient conditions, they felt considerably closer to the experimenter than did participants in the “cold” condition. Similarly, Williams and Bargh (2008) found that participants who were primed with physical sensations of warmth by holding a warm cup of coffee found an interaction partner to be a kinder person (see also Semin and Garrido, 2012, for similar findings).

Another related set of studies has suggested that certain movements are associated with warmth judgments. For example, Wentura et al. (2000) instructed participants to perform a lexical decision task on a set of stimuli by either pushing a button (an approach direction) or withdrawing their hand from a button (an avoidance direction). Results showed that participants were faster to press a button for positive warmth stimuli than for negative ones. The reverse was found when participants were withdrawing the hand from the button. That is, responses were faster for negative warmth stimuli than for positive ones. In a recent study, Freddi et al. (2014) demonstrated that warmth traits were judged more positively when these were moving on the screen toward the person rather than away from the person. In sum, this evidence suggests that approach-avoidance dimension might be a way of embodying warmth concepts.

Other studies have demonstrated that such non-verbal behaviors of competence as dominance and power (Cuddy et al., 2011) can also be interwoven with action. One line of research has suggested that vertical spatial dimension is associated with power. In these studies, participants were asked to judge concepts related to power that appeared either in the upper or lower half of the computer screen. Those who judged words in the upper half of the screen (i.e., power is UP metaphor) identified the words more quickly and more accurately than did those who judged the words in the lower half of the screen. Similar results were obtained when participants were asked to identify “powerful” words with up or down movements (e.g., Schubert, 2005; Giessner and Schubert, 2007).

Even more importantly, another line of research directly related to this work has suggested a spatial mapping of such competence-related concepts as power and achievement onto forward movements. With regard to power, Maner et al. (2010) found that priming the concept of power had an effect on forward action initiation. With regard to achievement, Natanzon and Ferguson (2012) showed that when participants were primed with forward movement, they demonstrated greater implicit positivity toward the concept of achievement. In the same vein, Landau et al. (2014) demonstrated that when participants imagined themselves on a forward path through college, their motivation for goal-directed actions increased. Finally, another recent study of Robinson and Fetterman (2015) argued that success can also be understood via forward movements through space. Thus, these studies strongly suggest that competence is at least partially expressed through forward bodily action.

## Integration of both theories

The current research builds on studies which found that metaphoric expressions such as “grasp the concept” were understood faster when they were preceded by either actual or imagined forward action that matched the direction of action implied by a metaphorical phrase (e.g., Gibbs, 2006; Wilson and Gibbs, 2007, for a review). These studies argue that people's posture and physical action influence processing of the metaphor as performing or imagining doing the body action facilitates construal of the abstract concept as a physical entity. This suggests that metaphorical conceptualization is embodied in the sense that when participants read a sentence in which a politician says “We will move toward a Great Society,” they actually imagine traveling along a physical path in order to reach their goal. In addition, the idea that comprehending language about actions requires the recruitment of motor planning systems is also supported by neuroscience literature which showed differential activation of leg muscles when reading sentences about leg actions (e.g., Buccino et al., 2003). Yet, one should raise the question whether perceived similarity between the self and the speaker (i.e., a politician), caused by shared experiences of either real or imagined forward movement, may cause interpersonal evaluative judgments.

A few sources of evidence point to the conclusion that bodily representations for self and the other may affect such judgments. First, neuroscientific research on the “mirror neuron system” has shown that similar brain regions are activated when people observe bodily state in others or experience that bodily state themselves (e.g., Keysers and Gazzola, 2009). Second, consumer psychology research has demonstrated that perceived similarity between the self and the other increases the likelihood of feeling attraction and being persuaded by the communication (e.g., Han et al., 2007). Third, social psychology research on perspective taking (i.e., putting oneself in the shoes of another) conducted by Galinsky and colleagues revealed that when perspective-takers have a proclivity for thinking of themselves as smarter and well-intentioned, they seem to ascribe their positive self-descriptions to the target person or group (e.g., Galinsky and Moskowitz, 2000; Galinsky and Ku, 2004).

What are we to make of this body of information on connection between the self and the speaker if we place these findings alongside SCM evidence suggesting that such non-verbal behaviors as expansive (e.g., taking up more space) and open (e.g., keeping limbs open) body postures reflect people's feeling of competence (see Cuddy et al., 2011 for reviews)? Similarly, how is this self-speaker connection relevant to evidence in support of CMT suggesting that people's understanding of metaphorical phrases is based on simulations of metaphorical actions described in these phrases (see Gibbs, 2006, for reviews)? We interpret this body of information as indicative of a possible integration between the self-concept (i.e., self's power or dominance or, more generally, competence) embodied in forward motor representations and the processing of a GOAL-AS-JOURNEY metaphor. More specifically, in this article we predicted that because forward movements and approach-oriented postures enhance people's competence-related self-conceptions and because the processing of the GOAL-AS-JOURNEY metaphor is facilitated by compatible forward movements, the self is associated with another individual through the construction of embodied simulations related to mentioned actions, which lead to a perceived similarity between the self and the speaker. These embodied simulations entail a transfer of competence-related self-conceptions from the self to the other and thus should affect the judgments of the other individual.

## Overview of present research

Experiments 1 and 2 tested two predictions. First prediction was that overt forward bodily movement and approach-oriented posture would have an effect on task-related competence social judgments, given the perceived similarity between the self and the speaker. Second prediction was that a social concept of warmth would not be embodied in the journey metaphor because participants infer the meaning of the text by imagining their participating in forward movement actions that facilitate their metaphoric understanding of task-related purposes (e.g., we will break the wall of hesitation and safely navigate our vessel to the better future) rather than relation-oriented attitudes (e.g., this politician is friendly, helpful, etc.). To test these hypotheses, in two experiments participants were asked to read an adapted version of the speech of President Lyndon Johnson on envisions of transforming America into a “Great Society” (Torricelli and Carroll, 1999) while their body was prepared for the processing of action-congruent information.

The text in Experiment 1 contained metaphors and metaphorical phrases implying forward movements. Participants in the advanced facilitation condition first exercised on a stationary bike and then read the text standing in front of the monitor while their lead leg was advanced for 40 cm from their follow leg. Participants in the control condition read the text with their feet together. The text in Experiment 2 differed depending on participants' group. Half of the participants (in both experimental and control conditions) read the text identical to that of Experiment 1 and the other half (in both experimental and control conditions) read the text that did not contain metaphorical statements as these were replaced by non-metaphorical expressions. In the experimental condition participants read the text in the same way as participants from the advanced facilitation condition, that is, with their lead leg advanced (with the exception that they did not exercise on a stationary bike before reading the texts). Participants in the control group read the text with their feet together. Such a division of participants was done to test whether metaphor (i.e., metaphoric vs. non-metaphoric text), embodiment (i.e., physical posture in experimental vs. physical posture in control condition) or their interaction is influencing person perception. After reading the text, participants in all conditions completed a 44-item questionnaire. The first 24 items were filler items and the rest of the items were target measures that assessed politician's competence and warmth.

## Experiment 1

In Experiment 1 participants were asked to read the speech containing a metaphorical statement “making progress is forward movement” in which a politician describes the steps needed to achieve the task of building a better society. Participants in the experimental (advanced facilitation) condition first exercised on a stationary bike for a minute and then were asked to read the text standing with their lead leg advanced forward. We assumed that this approach-oriented posture manipulation would activate competence-related embodied self-conceptions (i.e., power, achievement, etc.) and simulate preparation for forward movement. Participants in the control condition read the text standing with their feet together. Importantly, standing posture was chosen as a comparison condition rather than seated (vs. forward) or leaning back (vs. forward) as we wanted to ensure that such potential confounding variables as the comfort or difficulty of the poses had no effect on the variables being studied.

After reading the text, participants in both conditions were asked to fill in the questionnaire that, among other things, assessed participants' judgment of the target individual's competence and warmth skills. Our expectation was that participants in the experimental condition should rate higher the competence of the politician compared to the participants in the control condition, precisely because of their matching body action (e.g., forward movement) that enhances people's embodied construal of abstract concepts. However, if embodied hypothesis is correct, we should find no difference between participants with regard to warmth dimension as a forward movement facilitates participant's access to their embodied understanding of progress related to task achievement rather than progress related to honesty or kindness of a person.

### Method

#### Sample size considerations

Sample size was determined a–priori using Gpower 3.1.9.2 (Faul et al., 2007) for *F* tests (ANOVA: Repeated measures, within-between interaction). As previous research with action simulation paradigms led us to expect medium effect sizes (for reviews, see Horchak et al., 2014b) the parameters were set as follows: effect size *f* = 0.25, alpha level = 0.05, and power = 0.80. The calculation suggested a minimum total sample size of 34.

#### Participants and design

The sample consisted of 40 native Portuguese-speaking university students (*M*_age_ = 22.87, *SD*_age_ = 5.43) who were randomly assigned to each of the conditions. Ten participants were male (*M*_age_ = 25.70; *SD*_age_ = 7.23), 28 were female (*M*_age_ = 22.00; *SD*_age_ = 4.60), and two participants did not indicate gender. The first order statistical analysis was a mixed model 2 (condition: advanced facilitation vs. control) × 2 (social judgment: competence vs. warmth) ANOVA, with the first factor manipulated between participants and the second factor manipulated within participants. The second order planned comparisons were *t*-tests.

### Materials

#### Text

A speech of President Lyndon Johnson “Great Society” (Torricelli and Carroll, 1999) was adjusted to meet research requirements (see Annex A in Supplementary Material). More precisely, adjustments to the original text were as follows: the size of the text was reduced to seven paragraphs and 12 metaphors (metaphorical phrases) implying forward movements were embedded into it (metaphorical phrases describing forward movements were in each paragraph of the text). Importantly, words “competent” and “warm” were never used in the text, thus reducing the possibility that these two concepts were semantically primed.

#### Questionnaire

##### Competence

The primary dependent measure was a social judgment toward the politician as a competent individual. This dependent measure was assessed using 13 items (see Table 1), which were averaged to form an index of competence (Chronbach's alpha = 0.94). More specifically, these items asked participants to indicate, on a 7-point scale (1, Don't agree at all; 7, Totally agree), the extent to which they believed a politician to be competent.

**Table 1:** **List of words used in a questionnaire to assess competence and warmth judgments**.

**Competence traits**	**Warmth traits**
Decisive	Friendly
Competent	Reliable
Independent	Kind
Ambitious	Well-intentioned
Capable	Enthusiastic
Persuasive	Sincere
Skillful	Tolerant
Creative	
Active	
Confident	
Dynamic	
Competitive	
Efficient	

##### Warmth

Although, we did not predict any role of metaphoric simulation for warmth judgments in the context of the present study, it was important to include this dimension to the analysis to rule out alternative explanations for the findings grounded in the existing literatures on attitudes, group stereotypes, and embodiment. Warmth was assessed using seven items (see Table 1), which were averaged to form an index (Chronbach's alpha = 0.85). The items asked participants to indicate, on a 7-point scale (1, Don't agree at all; 7, Totally agree), the extent to which they believed a politician to be kind and friendly.

In choosing the words to assess competence and warmth dimensions, we took our cue from the work of Fiske et al. (2007) and Wojciszke (2005) who have argued that competence and warmth underlie the content of most group stereotypes as well as from the research of Rosenberg et al. (1968) regarding a two-dimensional configuration of 60 traits which show the properties of social (i.e., warmth) and intellectual (i.e., competence) desirability.

### Procedure

All participants voluntarily took part in the experiment. Anonymous survey did not require a written consent form, but the survey form started with the same explanation of the research protocol that would be used for a written consent form. The participant's decision to complete and return the survey implied consent to participate. All procedures were executed in compliance with relevant laws and institutional guidelines. The rights of student participants were protected under the institutional protocol called “The Letter of Rights and Responsibilities of Scientific Community” approved by the University of Algarve General Counsel on January 28th, 2013.

All participants were tested individually. Upon entering a quiet sound insulated room, they were ensured of the confidentiality of their responses and the possibility to stop participation whenever they want. First, participants were randomly assigned to one of two groups and asked to read the text on a laptop computer, which was placed on a height adjustable rostrum. More precisely, participants from the control group read the text while standing erect with the two feet together. Participants from the advanced facilitation condition were asked to pedal a stationary bike for a minute (resistance on pedals was set to a minimum; participants pedaled at a normal cycling speed of 80 revolutions per minute) and then read the text while standing erect with their lead leg advanced forward (40 cm from the follow leg). Participants were told that the purpose of the bike exercise and lead leg manipulation was to investigate how enacting a particular body posture affects the ease of reading.

The purpose of manipulating dominant (i.e., lead) rather than non-dominant leg was to ensure maximum integratability between approach-oriented posture and simulation constructed of the content of metaphoric discourse. Importantly, to find out which leg was lead, prior to experiment participants orally informed us about their dominant hand (right- or left-handed). Additionally, following an advice of an athletic instructor who suggested that the dominant leg does not always match the dominant hand, participants in the advanced facilitation condition were requested to go over the low 20-cm hurdle to see what leg would go over first. This test was recognized as reliable in determining leg dominance (e.g., Porac and Coren, 1981). For all participants the dominant hand was consistent with the lead leg. Importantly, participants in the control condition did not do the hurdle task.

Second, participants were asked to fill in a 44-item questionnaire. Consistent with the cover story, the first 24 items of the questionnaire were filler items. More precisely, participants were asked to report on a 7-point scale their perceived ease of reading (e.g., “I did not encounter any difficulties understanding the details of the text”), reading satisfaction (e.g., “I found the text easy to read”), vividness of mental imagery (e.g., “The images I formed in thinking about scenes were vivid-vague, clear-unclear, indistinct-distinct, sharp-dull, intense-weak, lifelike-lifeless, fuzzy-unambiguous”), and spatial presence (e.g., “I felt like I was part of the environment in the presentation”). These items were used to bolster the cover story as the whole premise of demonstrating the metaphoric construal of abstract concepts hinges upon participants not being aware that the postures and actions they are asked to perform represent metaphors. The suggested proportion of filler and experimental items reflects current methodological tendencies in embodiment research (see Wilson and Gibbs, 2007; Pecher et al., 2009, for a similar proportion of filler and experimental stimuli). The rest of the items were target items which asked participants to rate the competence and warmth qualities of the politician described in the text. Importantly, participants in both conditions filled in the questionnaire, which was placed on a height adjustable rostrum, standing with the two feet together. Finally, participants were debriefed if they suspected the real purpose of the manipulation procedure. More precisely, they were asked whether they saw any relation between the questionnaire measurements and body posture (and bike exercise in the case of Experiment 1), or noticed anything unusual. This was done to remove the data from those who would answer in the affirmative.

### Results

#### Initial data analysis

Before analyses, all variables were examined for normality. To minimize the influence of outliers, an outlier labeling rule was applied which is based on finding the difference between the first and third quartile of the distribution and multiplying it by a parameter, *g* = 2.2 (Hoaglin et al., 1986; Hoaglin and Iglewicz, 1987). Several outliers were noted (nine variables in experimental condition and five variables in control condition). Rather than eliminating them from the analyses, we winsorized them by replacing the top and bottom 2.5% of the cases on each variable with the value of their respective 2.5 and 97.5th percentile. This procedure has been recommended as effective in reducing the influence of outliers on variance estimates of standard error (e.g., Erceg-Hurn and Mirosevich, 2008). Overall, this procedure affected less than 2% of the data.

Data distribution through significance tests showed that the distribution of scores did not significantly differ from a normal distribution (both Kolmogorov-Smirnov's and Shapiro-Wilk's tests showed the values >0.05). Furthermore, a visual inspection of P-P plots and Q-Q plots indicated that all variables appeared to approximate normal distribution. Similarly, preliminary analyses of homogeneity of variance showed that the spread of scores was roughly equal in different group of cases (values from Levene's test were more than 0.05).

Finally, preliminary analyses of variance showed that there was no significant main effect of gender and no interaction between social judgments and gender, suggesting that the ratings from male and female participants were in general the same (all *p*s > 0.10). Debriefing showed that none of the participants was aware of the true purpose of the experiment. For the analyses below, all effects are reported as significant at *p* < 0.05.

#### Main analysis

**Figure 1:**
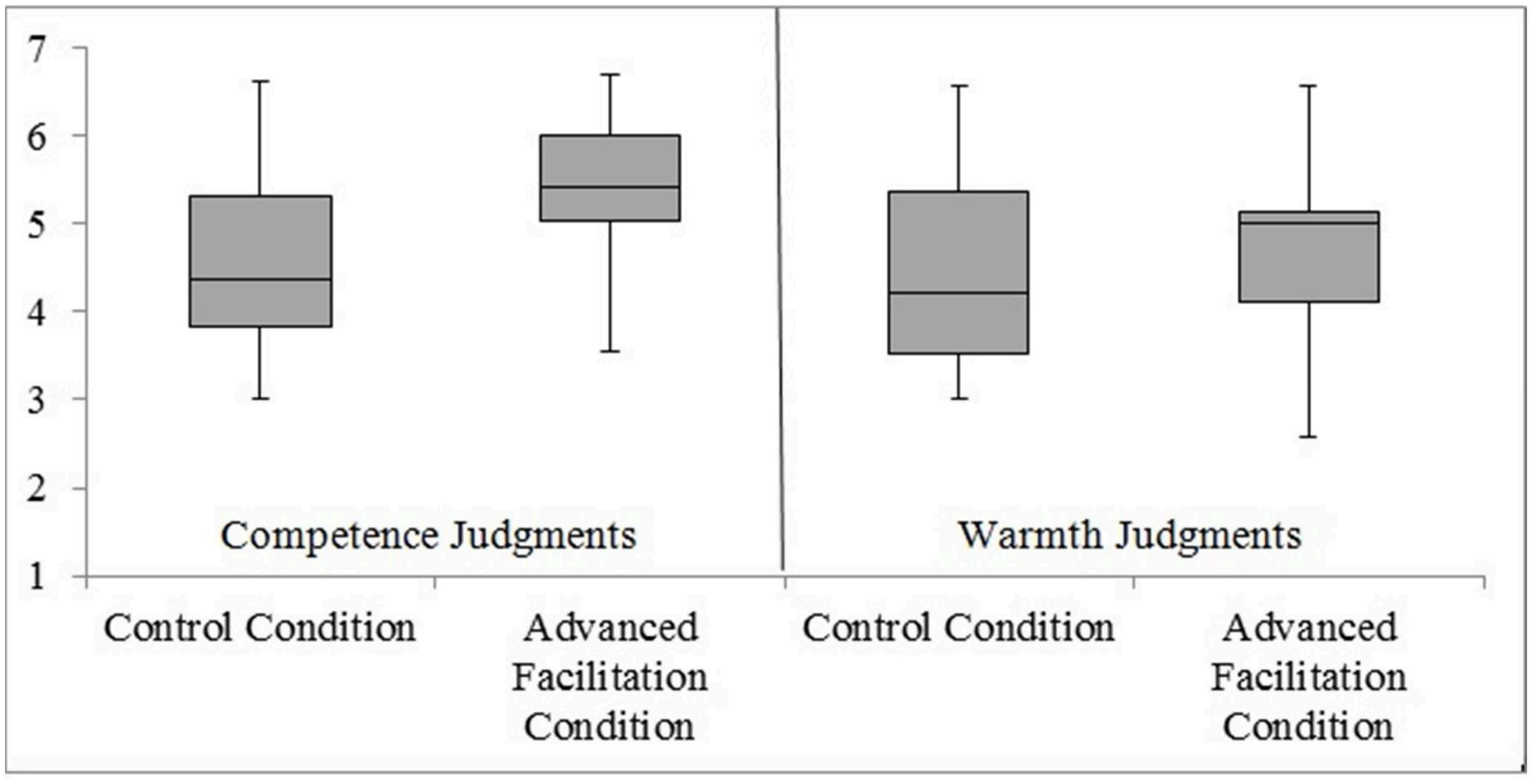
**Experiment 1: A Box Plot displaying how participants ascribed competence and warmth characteristics to a politician in control and advanced facilitation conditions**. At the center of the plot is the median, which is surrounded by a box the top and bottom of which are the limits within which 50% of observations fall. The whiskers at the top and bottom represent the most and least extreme scores, respectively.

The most important data are presented in Figure 1. Most critical to our predictions was a significant interaction between personality dimension and condition, *F*_(1, 38)_ = 4.53, *p* = 0.04, η^2^_*p*_ = 0.106. To break down this interaction, second order planned comparisons (*t*-tests) were performed comparing each social judgment (competence vs. warmth) across advanced facilitation and control conditions. These revealed that participants who engaged in body movements (experimental advanced facilitation condition) ascribed more competence (*M* = 5.44; *SD* = 0.80) to a politician than did participants who did not engage in such movements (control condition; *M* = 4.64; *SD* = 1.12), *t*_(38)_ = −2.61, *p* = 0.013, *r* = 0.39. In contrast, the index for warmth traits did not differ significantly between the experimental (*M* = 4.66; *SD* = 1.11) and control (*M* = 4.48; *SD* = 1.16) groups, *t*_(38)_ = −0.51, *p* = 0.618, *r* = 0.08. In addition, there was a significant main effect of social judgment, *F*_(1, 38)_ = 10.56, *p* < 0.01, η^2^_*p*_ = 0.217.

### Discussion

Experiment 1 was designed to test whether enhancing people's embodied, metaphorical construal of abstract concepts such as “making a progress is forward movement” has any effect on social judgments related to competence. Our findings provide initial support for the hypothesis that because in the event-structure metaphor “purposes are destinations to be reached” (Lakoff and Johnson, 1980), there exists a link between the content of the competence concept and a metaphoric, embodied construal of abstract entities at the level of the motor system. More precisely, we found that the embodied competence-related self-conceptions expressed and reinforced through forward movements and approach-oriented postures were associated with a politician through the construction of embodied simulations referred to in metaphorical expressions. Competence evaluations were influenced by compatible body action because embodied simulation entails a transfer of competence-related self-conceptions from the self to the other. Finally, consistent with a prediction, enhancing people's metaphoric construal of “progress” metaphor had no effect on warmth judgments as such judgments are typical of those who have a proclivity for relation-oriented attitude rather than task-oriented attitude (Wojciszke, 2005).

One could argue, however, that the observed significant effects are not a product of embodiment *per se*, but of disparity in the nature of the two social judgments, thus suggesting that competence and warmth are quite different in their metaphoricity (i.e., power of metaphor) alone. In other words, warmth is directly related to a metaphoric source domain (“Affection is Warmth”), while competence does not seem to be directly related to a specific metaphoric source domain. Similarly, it could be argued that differences between responses of experimental and control groups with regard to competence judgments are due to basic associative processes (see Briñol and Petty, 2008, for further discussion and review of relevant findings). More precisely, it is possible that participants in the experimental condition evaluated the competence characteristics of the politician higher because forward movement is already associated with progress, thus suggesting that the compatibility between real body action and that implied by metaphorical phrases is not necessary. To help rule out this concern, in Experiment 2 we made a direct contrast between the competence and warmth judgments in the experimental and control conditions by asking half of the participants to read a metaphoric text and the other half—a non-metaphoric text.

## Experiment 2

The purpose of Experiment 2 was two-fold. The first aim was to replicate and extend the findings from Experiment 1. In Experiment 2 participants in the experimental (basic facilitation) condition did not exercise on a bike prior to text reading. Thus, we could determine if the mere adoption of body posture appropriate for metaphorical phrases would have an effect on task-related competence social judgments. If the effect in Experiment 1 is replicated, it would lend more credence to our argument that there is a link between the content of the competence concept and an embodied construal of abstract entities at the level of the motor system. Furthermore, this additional evidence would support recent research in the area of language processing suggesting that even imagined action (Wilson and Gibbs, 2007; Gibbs, 2013) can enhance the embodied construal of metaphorical concepts as effectively as an actual body movement. The second goal was to test directly whether metaphor, embodiment, or their interaction is influencing person perception and rule out alternative associative-based explanations. To this end, we randomly split participants into two equal groups and asked them to read a political speech either in advanced facilitation or control condition. But whereas participants from one group read the text identical to that in Experiment 1, the participants from the other group read the same text as in Experiment 1, but this time metaphorical phrases implying forward movements were replaced by non-metaphorical expressions. The prediction derived from the embodied theory is that with regard to this latter group there will be no embodied effect of body posture on person perception due to the lack of integratability between body action and non-metaphoric discourse content.

### Method

#### Sample size considerations

Sample size was determined a–priori using Gpower 3.1.9.2 for *F* tests (ANOVA: Repeated measures, within-between interaction). As previous research with action simulation paradigms led us to expect medium effect sizes (for reviews, see Horchak et al., 2014b) the parameters were set as follows: effect size *f* = 0.25, alpha level = 0.05, and power = 0.80. The calculation suggested a minimum total sample size of 48. We tested more participants than the power analyses suggested as we aimed to have similar sample sizes in each condition across both experiments.

#### Participants and design

The sample consisted of 76 native Portuguese-speaking university students (*M*_age_ = 23.79, *SD*_age_ = 5.79) who were randomly assigned to each of the conditions. Twenty nine participants were male (*M*_age_ = 26.10; *SD*_age_ = 6.59) and 47 were female (*M*_age_ = 22.36; *SD*_age_ = 4.78). The first order statistical analysis was a mixed model 2 (condition: basic facilitation vs. control) × 2 (text type: metaphoric vs. non-metaphoric) × 2 (personality dimension: competence vs. warmth) ANOVA, with the first two factors manipulated between participants and the last factor manipulated within participants. The second order planned comparisons were *t*-tests.

### Materials and procedure

All participants voluntarily took part in the experiment. Anonymous survey did not require a written consent form, but the survey form started with the same explanation of the research protocol that would be used for a written consent form. The participant's decision to complete and return the survey implied consent to participate. All procedures were executed in compliance with relevant laws and institutional guidelines. The rights of student participants were protected under the institutional protocol called “The Letter of Rights and Responsibilities of Scientific Community” approved by the University of Algarve General Counsel on January 28th, 2013.

The same questionnaire items as in Experiment 1 were used. The text was the same, except that half of the participants (38 participants) in both control and advanced facilitation conditions read the political speech in which metaphorical phrases implying forward movements were replaced by non-metaphoric words or expressions (see Annex B in Supplementary Material). Procedure was nearly identical to that in Experiment 1, except that participants in the basic facilitation condition did not exercise on a bike before reading the text. Importantly, however, these participants did the hurdle task. Otherwise, the experiment was substantially identical to Experiment 1.

### Results

#### Initial data analysis

An outlier identification procedure with *g* parameter set to 2.2 did not find any values that exceed the boundaries of the “true” distribution, and hence no data were winsorized.

Data distribution through significance tests showed that the distribution of scores did not significantly differ from a normal distribution (both Kolmogorov-Smirnov's and Shapiro-Wilk's tests showed the values >0.05). Furthermore, a visual inspection of P-P plots and Q-Q plots indicated that all variables appeared to approximate normal distribution. Similarly, preliminary analyses of homogeneity of variance showed that the spread of scores was roughly equal in different group of cases (values from Levene's test were more than 0.05).

Preliminary analyses of variance showed that there was no significant main effect of gender and no interaction between social judgments and gender, suggesting that the ratings from male and female participants were in general the same (all *p*s > 0.10). Debriefing showed that none of the participants was aware of the true purpose of the experiment.

#### Main analysis

Of main interest to our hypothesis was a significant interaction among the type of social judgment (competence vs. warmth) × condition (control vs. basic facilitation) × text type (metaphoric vs. non-metaphoric), *F*_(1, 72)_ = 5.04, *p* = 0.028, η^2^_*p*_ = 0.065. Contrasts were used to break down this interaction.

**Figure 2:**
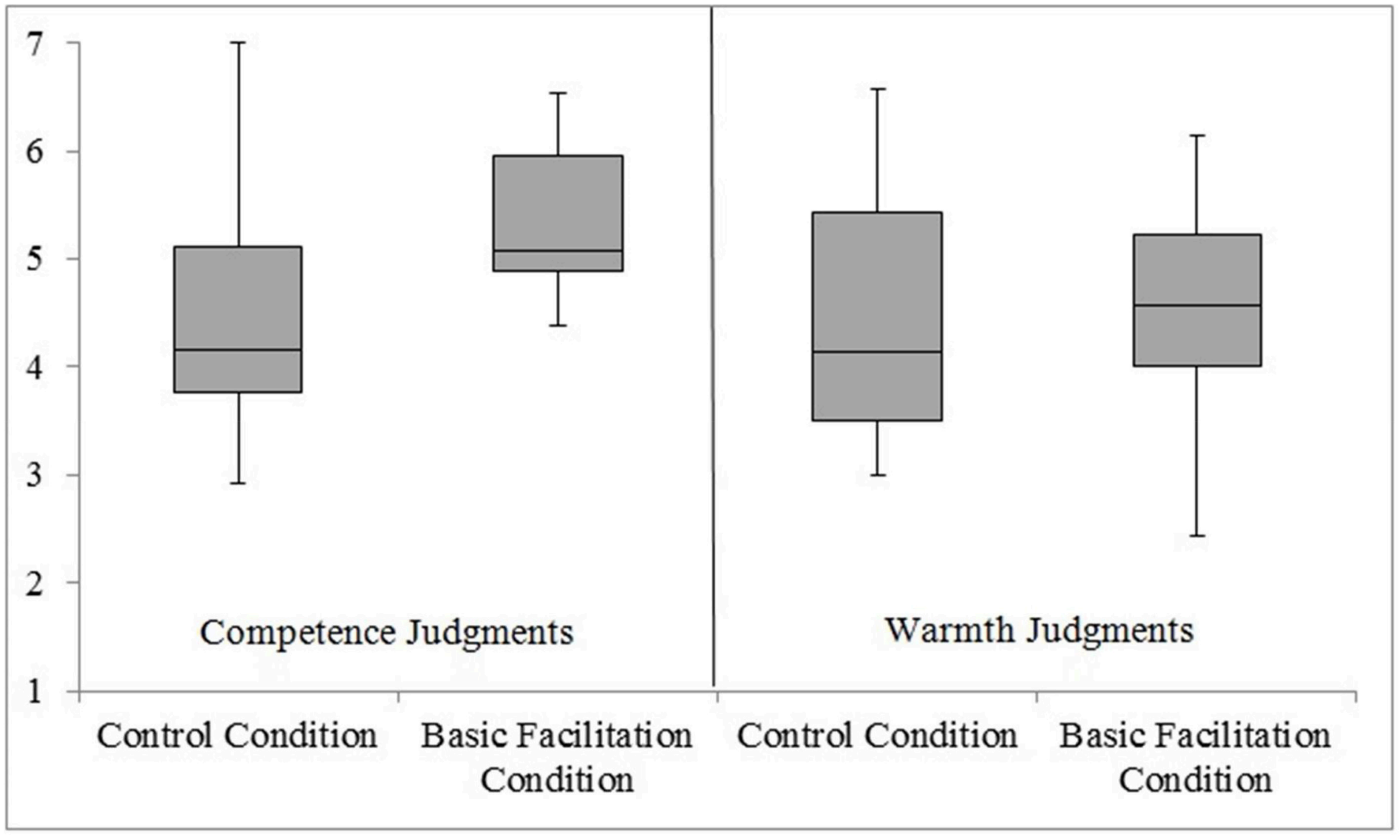
**Experiment 2 (Metaphoric Text): A Box Plot displaying how participants ascribed competence and warmth characteristics to a politician in control and basic facilitation conditions**. At the center of the plot is the median, which is surrounded by a box the top and bottom of which are the limits within which 50% of observations fall. The whiskers at the top and bottom represent the most and least extreme scores, respectively.

The first contrast compared the scores of participants from control and advanced facilitation conditions at each level of social judgment (competence vs. warmth) when the metaphoric text was read. Similar to Experiment 1, *t*-tests showed that participants whose bodily systems were prepared for forward movement associated with metaphorical phrases (experimental basic facilitation condition; *M* = 5.40; *SD* = 0.67) ascribed more competence to a politician than did participants who did not prepare for such movements (control condition; *M* = 4.55; *SD* = 1.19), *t*_(36)_ = −2.71, *p* = 0.010, *r* = 0.29. In contrast, the index for warmth traits did not differ significantly between the experimental (*M* = 4.54; *SD* = 0.95) and control (*M* = 4.44; *SD* = 1.18) groups, *t*_(36)_ = −0.27, *p* = 0.79, *r* = 0.13 (see Figure 2).

**Figure 3:**
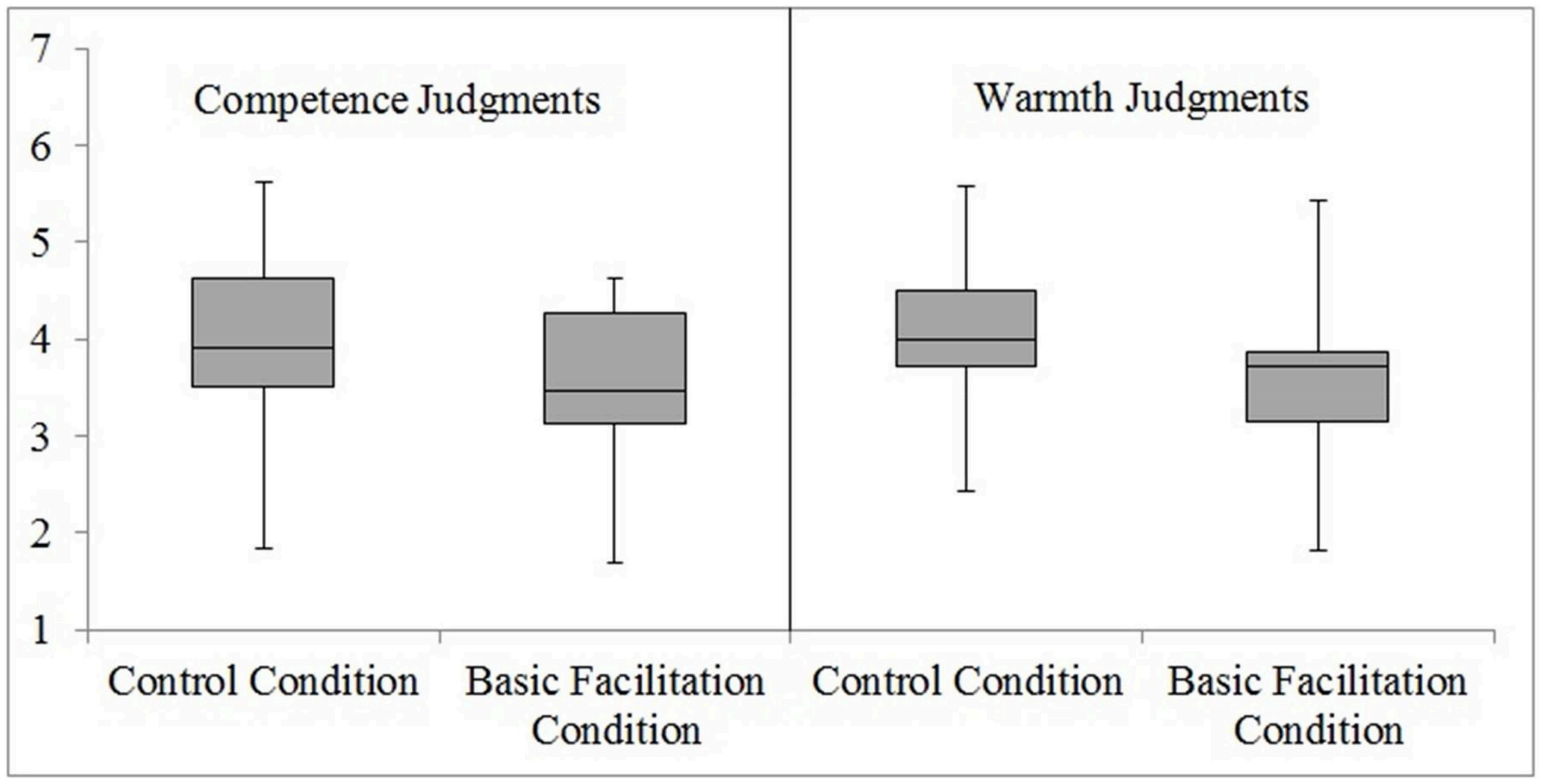
**Experiment 2 (Non-metaphoric Text): A Box Plot displaying how participants ascribed competence and warmth characteristics to a politician in control and basic facilitation conditions**. At the center of the plot is the median, which is surrounded by a box the top and bottom of which are the limits within which 50% of observations fall. The whiskers at the top and bottom represent the most and least extreme scores, respectively.

The second contrast compared the scores of participants from control and basic facilitation conditions at each level of social judgment (competence vs. warmth) when the non-metaphoric text was read. Unlike in the first contrast, *t*-tests showed that participants' scores did not differ significantly at each level of social judgment after reading the non-metaphoric text. More precisely, we found that participants whose bodily systems were prepared for movement (experimental basic facilitation condition; *M* = 3.55; *SD* = 0.77) did not significantly differ in their responses from participants in no movement condition (control condition; *M* = 4.04; *SD* = 0.94) regarding competence judgments, *t*_(36)_ = 1.77, *p* = 0.086, *r* = 0.28. The similar pattern of results was observed with regard to warmth judgments when comparing the scores of participants in basic facilitation (*M* = 3.57; *SD* = 0.77) and control conditions (*M* = 4.03; *SD* = 0.83), *t*_(36)_ = 1.77, *p* = 0.085, *r* = 0.28. It is interesting to note, however, that although second contrast was not significant, the difference in responses showed a trend toward higher perceived politician's competence for participants from the control condition (see Figure 3).

There were several other significant effects in the analyses of participants' mean ratings. There was a significant interaction between social judgment (competence vs. warmth) and text type (metaphoric vs. metaphoric), *F*_(1, 72)_ = 7.81, *p* = 0.007, η^2^_*p*_ = 0.098. This interaction indicates that the desirability of different character traits (competence vs. warmth) differed according to the type of text (metaphoric vs. non-metaphoric) participants were asked to read. To break down this interaction, contrasts were performed comparing each personality dimension to the category of text type. These revealed that participants who read a metaphoric text (*M* = 4.97; *SD* = 1.04) ascribed more competence to a politician than those participants who read a non-metaphoric text (*M* = 3.79; *SD* = 0.88), *t*_(74)_ = 5.31, *p* = < 0.001, *r* = 0.53. Participants who read a metaphoric text (*M* = 4.49; *SD* = 1.06) ascribed more warmth to a politician than those participants who read a non-metaphoric text (*M* = 3.79; *SD* = 0.88), *t*_(74)_ = 3.17, *p* = 0.002, *r* = 0.35. Finally, there was a significant main effect of personality dimension, *F*_(1, 72)_ = 7.49, *p* < 0.01, η^2^_*p*_ = 0.31.

### Discussion

The results from Experiment 2 accomplished three objectives. First, they demonstrated that the effect of enhanced embodied construal of the “progress” metaphor on social judgments related to competence can be observed even in a task with no real body movement. Second, they showed an interaction between metaphoric language use (i.e., metaphoric text vs. non-metaphoric text) and physical posture (i.e., basic facilitation vs. control). Thus, basic associative processes are unlikely to explain the current pattern of results. Third, the claim that significant effects may be explained by disparity in the nature of the two social judgments is not supported by the current data. We found that participants who read a metaphoric text ascribed both more competence and more warmth to a politician than participants who read a non-metaphoric text. This significant effect of text type means that if we ignore the condition in which participants read the text, then the ratings of politician's competence and warmth in the metaphoric text condition were in general the same. Thus, in the context of the present study we do not have good reasons to think that competence and warmth were quite different judgments in their metaphoricity alone.

## General discussion

The current research considered the question of how performing an action, or merely preparing the body for action, can have an impact on social judgments related to person perception. Two studies provide support for the conclusion that forward body movements enhance the favorability of competence judgments when these match the metaphoric forward movements described by text. Furthermore, when these findings are placed alongside evidence suggesting that body action facilitates construal of metaphorical abstract concepts as physical entities (e.g., Wilson and Gibbs, 2007; Gibbs, 2013), they suggest that such judgments are partially affected by concurrent kinesthetic experiences. More precisely, Experiment 1 showed that participants whose body action matched the metaphoric action described in the text ascribed more competence characteristics to a politician than did control participants. Experiment 2 demonstrated that participants whose body was merely prepared for forward movement also ascribed more competence characteristics to a politician than did control participants. In addition, the data from Experiment 2 ruled out an alternative non-embodied explanation (i.e., that effect is due to basic associative processes) grounded in the existing literatures on attitudes by demonstrating that body manipulation had no effect on competence when a non-metaphoric text was used. Finally, no evidence was found that body manipulation affects warmth judgments.

The present research is important for several reasons. First, it directly assesses the embodied metaphoric transfer effects on such non-observable characteristic of an individual as competence. More precisely, two studies show that perceptions of movement influence perceptions of individual's competence in line with their metaphoric relation (more competent is engaged in a purpose-based journey/less competent is not engaged in a purposed-based journey). These findings suggest that our understanding of competence is at least partially grounded in the bodily systems of action. This conclusion fits with embodied approaches to cognition (e.g., Lakoff and Johnson, 1980; Glenberg and Kaschak, 2002; Glenberg and Gallese, 2012) suggesting that the brain systems responsible for motor planning and execution influence the activation of target concepts in cognition. Another important contribution of this study is that it sheds light on the interplay of motor behavior and embodied metaphoric mapping and their potential impact on cognitive processing. Indeed, the reported results hint at the possibility that such abstract concept as competence may be partially understood via forward movements of the human body. Finally, our findings are consistent with the previous studies linking movement with activation of particular concepts. For instance, in research on metaphor's role in perceptions of social symbols and environments, Schubert (2004) showed that producing such body movement as making a fist activates the concept of social power. In the domain of language processing, Horchak et al. (2014a) demonstrated that individuals are faster in their judgments times with regard to explicit and implicit comprehension questions when their bodily systems are prepared for the processing of action-congruent information. Finally, Gibbs (2013) in a series of studies revealed that real body actions related to the actions mentioned in the narrative (e.g., walking while reading a sentence “moving along in a good direction”) have an effect on how people conceive of such abstract concept as relationship.

It is also worth noting that our results resemble some similarities to the metaphoric framing model which studies metaphor effects within the domain of political cognition (Ottati et al., 2014). According to this model, the processing of political discourse activates a root metaphor (e.g., Making a progress is a forward movement) which in turn connects the focus of the message (e.g., Making a progress) to the metaphorical source (e.g., “a forward movement”). In this way, metaphor allows people to draw on informational implications of the metaphorical source (e.g., forward movement is associated with goals, achievement, competence, etc.) as a framework for understanding the target (see also Landau et al., 2010, for a related discussion).

Although, our data show that bodily processes compatible with metaphoric language can affect social judgments related to competence, we do not claim that perceived competence is affected only by forward movements of the human body. Rather, we support the claim put forward by embodied scientists (e.g., Glenberg, 1997; Barsalou, 1999) suggesting that various patterns of perceptual and action interactions contribute to our concrete understandings of the abstract concepts. For example, people may as well ascribe more competence characteristics to individuals who actively gesticulate with their arms and hands while speaking, compared to those who don't gesticulate (see also, Pouw et al., 2014, for a discussion regarding cognitive function of gestures). In this manner, various bodily experiences (i.e., forward walking, gesticulation, etc.) provide the source concrete domains for grounding target abstract domains (Gibbs, 2006).

It is interesting to note that body manipulation tended to “impair” competence judgments when a non-metaphoric text was used. One possible explanation is the lack of integratability between the real motor action and the events described in discourse. For example, making a forward movement can be integrated with the content of the metaphoric sentence, “We will walk into the better future together,” whereas the same forward movement cannot be easily integrated with the content of the non-metaphoric sentence, “We will change the future together.” Another explanation is consistent with the metaphoric fit hypothesis (Keefer et al., 2014) which suggests that people are more favorable toward solutions when they accord with the metaphoric understandings of the problem. That is, if people represent progress metaphorically as forward movement and a politician fails to frame progress metaphorically as being spatially mapped onto forward movements, then he/she may be evaluated negatively on the subject of progress.

There are a few limitations of our experiments and caveats to our findings. The first limitation is generalizability, given that our experiments are limited to only one speech. It is unclear whether results are generalizable to other texts, but future research could seek to replicate the present findings using other speeches where the GOAL-AS-JOURNEY metaphors are used.

The second limitation of the present research concerns the way action system was manipulated in Experiments 1 and 2. It could be argued that the absence of the bike-alone task (note that lead leg manipulation was used in Experiments 1 and 2) does not allow drawing definite conclusions with regard to what contributed more to the construction of the simulation, lead leg manipulation (i.e., preparation for movement) or bike exercise (i.e., activation and engagement of the motor system). We did not include the bike-alone task as we suspected that such a manipulation would not be sufficient (though necessary) to significantly boost the construction of the action-based simulations. Our suspicion is supported by previous research (e.g., Stanfield and Zwaan, 2001; Zwaan et al., 2002, 2004; Kaschak et al., 2005; Yaxley and Zwaan, 2007) that stressed the importance of maximum integratability between the internal symbol and external referent (see also Barsalou, 1999, for the discussion of the analogous relationship between symbol and referent in perceptual symbols system). For instance, understanding the sentence, “We will altogether MAKE A GIANT STEP” requires the construction of the simulation that includes various perceptual symbols (e.g., a subject walking, a path, a distance between a lead leg and a follow leg, a direction of movement, etc.). This in turn leads to predictions about the interactions of certain characteristics of real-world movement and that described in the metaphoric phrase. Specifically, since the simulation should represent all possible physical characteristics of the object, it is reasonable to expect that the bike alone task is not enough to have a significant effect on processing of the phrase MAKE A GIANT STEP, given that this task shares content with the simulation of the phrase only with regard to such perceptual symbol as direction. Thus, it seems that the most successful simulation would arise from either lead-leg manipulation alone or two manipulations used together as these kinds of action manipulations would share content with the simulation of the phrase with regard to all of the above-mentioned perceptual symbols. Nevertheless, we acknowledge that a precise metric of integratability between a bike exercise and a simulation constructed of the content of text can only be provided by running an experiment with a bike-alone task. Additional research, such as asking participants to actually walk while they are listening to the text over headphones or read a text while walking on a treadmill would be invaluable to further test the predictions regarding integratability.

The third limitation of the present research concerns the procedural detail regarding a hurdle. It could be argued that this task led to confounds between posture and arousal with regard to participants in the facilitation condition. While such a possibility exists, we believe this is not the case for two reasons. First, we did not use a race hurdle over which participants had to leap. Note that the height of our hurdle was only 20 cm. Second, this procedure took less than 5 s as it only served to determine participants' lead leg.

To sum up, the present studies are the first to our knowledge to test the idea how constructing a simulation from a metaphoric language using the journey schema can influence our opinions about the competence of political leaders. They complement previous research on how relevant metaphors and embodied simulation can influence person perception and highlight the importance of embodiment for social cognition. Our findings also have important implications for political rhetoric. We propose that metaphors of movement may be useful not only for short-term objectives such as accomplishing a transfer of message that supports the goal of the politician, but also for long-term objectives such as shaping the image of politician as a competent leader. From a practical standpoint, this research suggests that the preparation of political speech targeted toward certain groups of people may experience greater or lesser success in shaping politician's image depending on the type of metaphor used in the speech. We hope that this study may provide knowledge for political image makers to make politicians look good and help them build support for worthwhile policies.

## Author contributions

Conceived and designed the experiments: OH, JG. Performed the experiments: OH. Suggested questionnaire items: JG. Suggested statistical procedures: OH, JG, MG. Analyzed the data: OH. Contributed to the interpretation of data for the work: OH, JG, MG. Drafted the work: OH. Critically revised the work: OH, JG, MG. Approved the revision to be published: OH, JG, MG.

## Funding

This research was supported by Grant EMECW-LOT 7 (Application no. 47), implemented by the Education, Audiovisual, and Culture Executive Agency of European Commission, awarded to OH; Grants FP7-PEOPLE-2013-CIG and PTDC/MHC-PCN/5217/2014 awarded to MG, and partially financed by the Foundation for Science and Technology (Portugal) through CIEO fund PEst-OE/SADG/UI4020/2014.

### Conflict of interest statement

The authors declare that the research was conducted in the absence of any commercial or financial relationships that could be construed as a potential conflict of interest.
