# Supplementary material for: Action Contribution to Competence Judgments: The Use of the Journey Schema
Source: Front Psychol. 2016 Mar 30;7:448. doi: 10.3389/fpsyg.2016.00448 (PMC4812067; doi:10.3389/fpsyg.2016.00448)
Supplement: Supplementary file 1 [file DataSheet1.docx]

*Annex A*

A Complete Version of the Metaphoric Text “Great Society” used in Experiments 1 and 2

**“Great Society”**

By protecting the life of our nation and reserving the liberties of our citizens we pursue our own happiness. Our success in that pursuit is the test of our success as a nation. Your imagination, your initiative, and your creativity will help us make a society where the progress is the servant of our needs. For in your time we have the opportunity to move not only toward the rich society and the powerful society, but toward the Great Society.

If we have a false sense of independence, in the journey to the better tomorrow our ships can collide and crash. But if we find commitment to new priorities, to new strategies, and new ways of thinking that ensure that hope will be kept alive, we will break the wall of hesitation and safely navigate our vessel to the better future.

So I want to talk to you today about the places where we begin to build this Great Society: in our cities and in our classrooms. Many of you will live to see the day, perhaps fifty years from now, when our population and city land will double, and when we will altogether make a giant step in the direction of building homes, highways, and facilities equal to all those built since this country was first settled. So in the next 40 years we will be building the bridge to pave the path to this Great Society.

Our society will never be great until our cities are great. On the wings of time we are quickly approaching an era when imagination and innovation become the biggest priorities. Use your imagination and hope as weapons of our progress to the Great Society. As riders in the race do not stop short as they reach the goal, so do you. Go forward, stretch your arms to grasp any single idea that will lead us to the better tomorrow.

Our society will never be great until more than one quarter of our society have not finished high school. Each year more than 10,000 high school graduates with proven ability do not enter college because they cannot afford it. And if we cannot educate today’s youth, we cannot follow the future with confidence. This year I launched the campaign to build an educational system which grows in excellence as it grows in size. I face it, we still have many problems in our schools, but at the same time we moved from the road of discord to the road of agreement. While walking on this road we have to go beyond the curricula which are outdated and move along the stream of future to find new ways to stimulate the love of learning and the capacity for creation.

These are the two of the central issues of the Great Society. While our government has many programs directed at those issues, I do not pretend that we have the full answer to those questions. But I do promise this: We are going to assemble the best thought and the broadest knowledge from all over the world in order to walk into the better future together and open a new chapter in the history of our great nation.

*Annex B*

A Complete Version of the Non-metaphoric Text “Great Society” used in Experiment 2

**“Great Society”**

By protecting the life of our nation and reserving the liberties of our citizens we ensure our own happiness. Our success in that task is the test of our success as a nation. Your imagination, your initiative, and your creativity will help us make a society where the progress is the servant of our needs. For in your time we have the opportunity to not only be the rich society and the powerful society, but also the Great Society.

If we have a false sense of independence, in the desire to change the future we may get disappointed. But if we find commitment to new priorities, to new strategies, and new ways of thinking that ensure that hope will be kept alive, we will no longer hesitate and achieve great success in our objectives.

So I want to talk to you today about the places where we begin to build this Great Society: in our cities and in our classrooms. Many of you will live to see the day, perhaps fifty years from now, when our population and city land will double, and when we will altogether make a great progress in the direction of building homes, highways, and facilities equal to all those built since this country was first settled. So in the next 40 years we will be working hard to make this Great Society a reality.

Our society will never be great until our cities are great. Very soon imagination and innovation will become the biggest priorities. Use your imagination and hope as weapons of our progress to the Great Society. As riders in the race do not stop short as they reach the goal, so do you. Be active, be opened to accept any single idea that will ensure better life tomorrow.

Our society will never be great until more than one quarter of our society have not finished high school. Each year more than 10,000 high school graduates with proven ability do not enter college because they cannot afford it. And if we cannot educate today’s youth, we cannot be confident about our future. This year I launched the campaign to build an educational system which grows in excellence as it grows in size. I face it, we still have many problems in our schools, but at the same time we managed to overcome discord and reach an agreement. While working on this agreement we have to change the curricula which are outdated and adapt it to the demands of future generations to find new ways to stimulate the love of learning and the capacity for creation.

These are the two of the central issues of the Great Society. While our government has many programs directed at those issues, I do not pretend that we have the full answer to those questions. But I do promise this: We are going to assemble the best thought and the broadest knowledge from all over the world in order to change the future together and mark a turning point in the history of our great nation.
